# Indoleamine 2, 3-dioxygenase 1 inhibitory compounds from natural sources

**DOI:** 10.3389/fphar.2022.1046818

**Published:** 2022-11-04

**Authors:** Ying Tan, Miaomiao Liu, Ming Li, Yujuan Chen, Meng Ren

**Affiliations:** ^1^ Experiment Center, Shandong University of Traditional Chinese Medicine, Jinan, China; ^2^ College of Pharmacy, Shandong University of Traditional Chinese Medicine, Jinan, China; ^3^ Office of Academic Affairs, Shandong University of Traditional Chinese Medicine, Jinan, China; ^4^ Second Affiliated Hospital, Shandong University of Traditional Chinese Medicine, Jinan, China; ^5^ United Front Work Department, Shandong University of Traditional Chinese Medicine, Jinan, China

**Keywords:** indoleamine 2, 3-dioxygenase 1, tryptophan, kynurenine, IDO1 inhibitors, natural compounds

## Abstract

*L*-tryptophan metabolism is involved in the regulation of many important physiological processes, such as, immune response, inflammation, and neuronal function. Indoleamine 2, 3-dioxygenase 1 (IDO1) is a key enzyme that catalyzes the first rate-limiting step of tryptophan conversion to kynurenine. Thus, inhibiting IDO1 may have therapeutic benefits for various diseases, such as, cancer, autoimmune disease, and depression. In the search for potent IDO1 inhibitors, natural quinones were the first reported IDO1 inhibitors with potent inhibitory activity. Subsequently, natural compounds with diverse structures have been found to have anti-IDO1 inhibitory activity. In this review, we provide a summary of these natural IDO1 inhibitors, which are classified as quinones, polyphenols, alkaloids and others. The overview of *in vitro* IDO1 inhibitory activity of natural compounds will help medicinal chemists to understand the mode of action and medical benefits of them. The scaffolds of these natural compounds can also be used for further optimization of potent IDO1 inhibitors.

## 1 Introduction

### 1.1 Tryptophan metabolism and indoleamine 2, 3-dioxygenase 1

**FIGURE 1:**
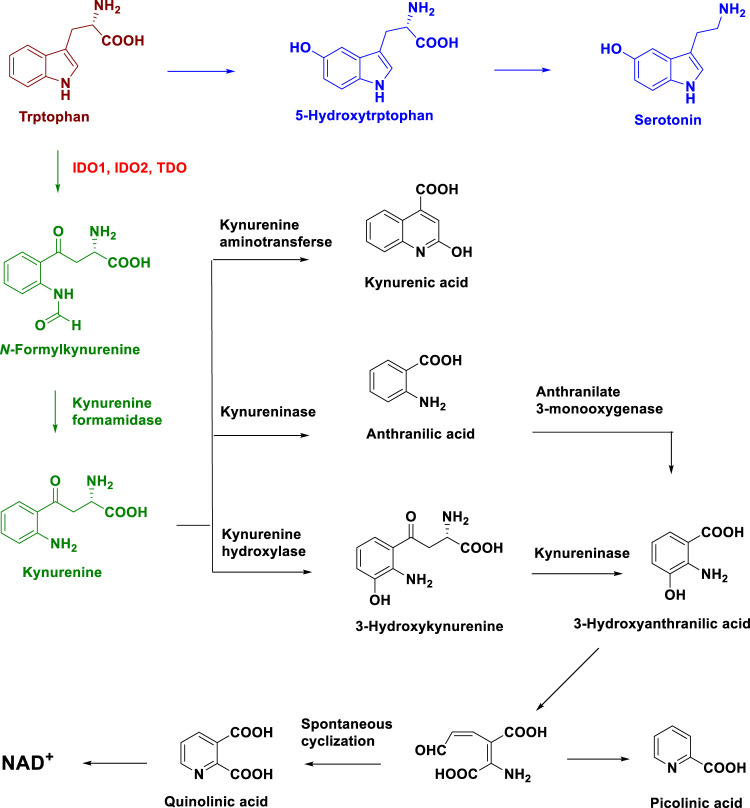
Metabolic pathway of *L*-Tryptophan.

*L*-Tryptophan (*L*-Trp) is an essential amino acid, and the normal concentration range of *L*-Trp in human plasma is 50–100 μM (Wang et al., 2015; Cervenka et al., 2017; Barreto et al., 2018; Platten et al., 2019). *L*-Trp is important as a protein building block and in the synthesis of several important bioactive metabolites (Wang et al., 2015; Cervenka et al., 2017; Barreto et al., 2018; Platten et al., 2019). However, humans cannot produce *L*-Trp and must obtain it from food (Barreto et al., 2018). The metabolism of *L*-Trp occurs *via* the serotonin pathway and kynurenine (Kyn) pathway (Figure 1) (Barreto et al., 2018). The Kyn pathway metabolizes 95% of *L*-Trp. In this pathway, *L*-Trp is oxidized, breaking the 2, 3-double bond of the indole ring to form *N*-formylkynurenine, which is rapidly converted to Kyn by Kyn formamidase. Next, Kyn is metabolized to kynurenic acid and 3-hydroxy-o-aminobenzoic acid, and 3-hydroxy-o-aminobenzoic acid is used to produce NAD^+^ (Figure 1). *L*-Trp consumption and Kyn production are key to immune system regulation under both physiological and disease conditions (Maddison and Giorgini, 2015; Barreto et al., 2018; Odunsi et al., 2022). Recent studies have continued to highlight the importance of *L*-Trp metabolism in immune regulation, neuronal function, and ageing (Sorgdrager et al., 2019; Platten et al., 2021; Krupa et al., 2022; Merlo et al., 2022; Ouyang et al., 2022; Salminen, 2022).

In the Trp to Kyn metabolic pathway, the oxidation of Trp to *N*-formylkynurenine is the initial and rate-limiting step, which can be catalyzed by the tryptophan dioxygenase isozymes indoleamine 2, 3-dioxygenase 1 (IDO1), indoleamine 2, 3-dioxygenase 2 (IDO2), and tryptophan 2, 3-dioxygenase (TDO) (Vécsei et al., 2013; Dounay et al., 2015; Dolšak et al., 2021). IDO1, IDO2, and TDO have differences in structure, tissue distribution, and substrate specificity. IDO1 is encoded by the *IDO1* gene on human chromosome eight and is widely present in the lung, intestine, colon, kidney, spleen, pancreas, central nervous system, macrophages, and microglia (Tone et al., 1990; Takikawa, 2005; Lewis-Ballester et al., 2017; Santos et al., 2022). IDO1 shows a broad substrate specificity for *L*-Trp (*K*_m_ = 20 μM), *D*-Trp, 5-hydroxy Trp, tryptamine, serotonin, and other Trp analogues (Pantouris et al., 2014). IDO2 is encoded by the *IDO2* gene on chromosome eight and is mainly distributed in the kidney, liver, and reproductive organs (Ball et al., 2007; Bakmiwewa et al., 2012; Fukunaga et al., 2012). The enzymatic activity of IDO2 is low, and the *K*_m_ of IDO2 for *L*-Trp is around 6.8 mM (Fukunaga et al., 2012). It is speculated that IDO2 might be more involved in cell signaling rather than functioning as a tryptophan dioxygenase (Fukunaga et al., 2012). TDO is encoded by the *TDO2* gene on chromosome four and is mostly distributed in the liver with highly specific enzymatic activity for *L*-Trp (*K*_m_ = 190 μM) (Löb et al., 2009; Pham et al., 2019). In summary, the three tryptophan dioxygenase enzymes show different substrate activities. The catalytic activity order of these three enzymes for *L*-Trp is IDO1 (*K*_m_ = 20 μM) > TDO (*K*_m_ = 190 μM) > IDO2 (*K*_m_ = 6.8 mM) (Dolšak et al., 2021).

**FIGURE 2:**
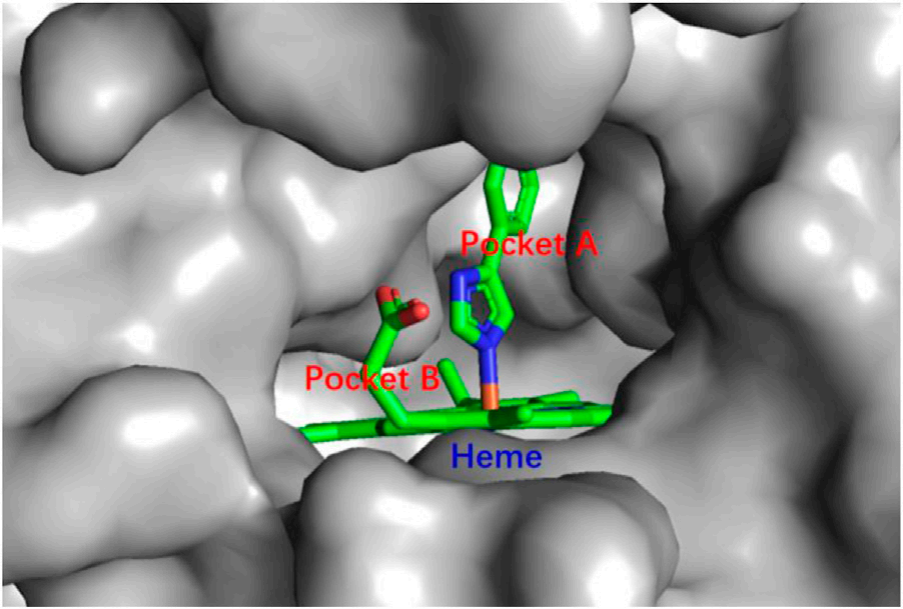
Crystal structure shows the active site of IDO1 with the binding of the ligand of PI.

IDO1 is a heme-containing enzyme composed of 403 amino acids. More than 60 human IDO1 crystal structures have been deposited in the Protein Data Bank (PDB) since the crystal structure of IDO1 was first reported in 2006 (PDB ID: 2D0T) (Sugimoto et al., 2006; Maddison and Giorgini, 2015). The crystal structure of IDO1 contains hydrophobic pockets A and B in the active site, with heme at the bottom of pocket A. The inhibitor ligand of PI was also included in the crystal structure (Figure 2) (Sugimoto et al., 2006). In addition, the JK loop forms the front entrance of the active site, which allows the substrate and inhibitors to enter (Lewis-Ballester et al., 2017). Interestingly, studies also revealed that the phosphorylation of two tyrosine residues of IDO1: Tyr115 and Tyr253 regulates the functions of this enzyme (Albini et al., 2017).

## 2 Indoleamine 2, 3-dioxygenase 1 and its functions

### 2.1 Immune tolerance in tumors

In tumor microenvironments, tumor killer cells, such as, effector T cells, natural killer cells, are often inhibited and induced to apoptosis to prevent the killer activity, and immune tolerance cells, such as, Treg cells, myeloid-derived suppressor cells, are often activated and promoted to the proliferation (Wu and Dai, 2017; Arneth, 2019). These combined effects create an immunosuppressive microenvironment suitable for tumor growth and lead to tumor immune escape. Research revealed that IDO1 is important in creating the immunosuppressive microenvironment (Ling et al., 2014; Munn and Mellor, 2016; Heidari et al., 2020; Gouasmi et al., 2022; Huang et al., 2022; Zhang et al., 2022). Under physiological conditions, IDO1 is usually expressed at a low level in various tissues. However, IDO1 is overexpressed in many cancers, such as, breast, colorectal, gastric, lung, and endometrial cancers, and IDO1 overexpression is also associated with poor survival rates (Uyttenhove et al., 2003; Dolusić et al., 2011; Heidari et al., 2020; Odunsi et al., 2022).

**FIGURE 3:**
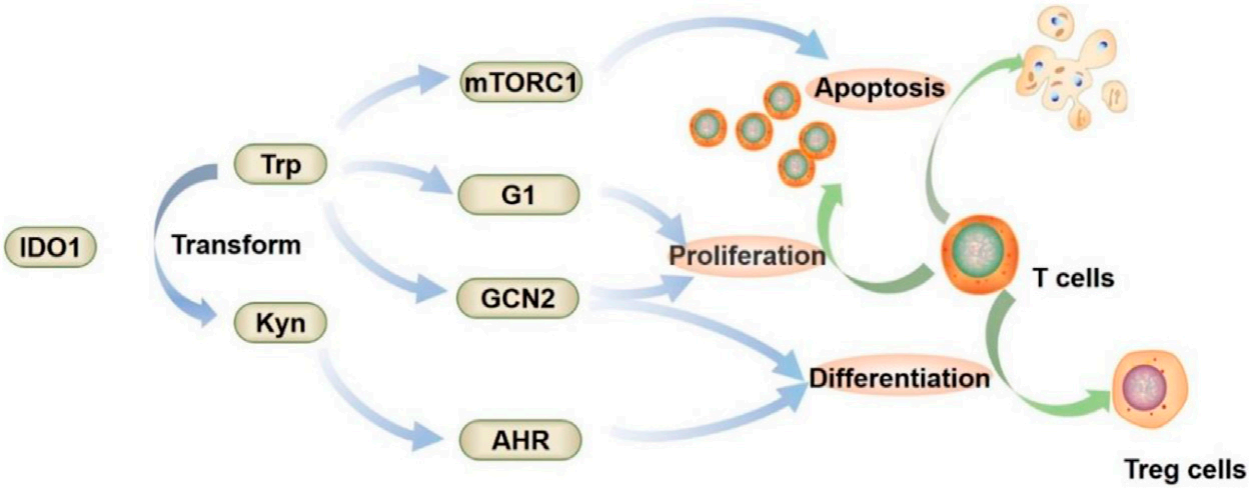
Mechanism of IDO1-mediated tumor immune escape.

IDO1 mediates tumor immune escape *via* three main downstream pathways (Figure 3) (Ling et al., 2014; Munn and Mellor, 2016; Heidari et al., 2020). In the first, tryptophan is depleted by the overexpression of IDO1, which increases the degradation of Trp and the production of Kyn. This causes the imbalance between Trp/Kyn in the tumor microenvironment (Liu and Wang, 2009; Zhai et al., 2020). The decrease in Trp and the increase in Kyn prevents T lymphocytes from maturing. IDO1 downstream metabolites, such as, Kyn, kynurenine acid (KA) and 3OH-Kyn, are toxic and can inhibit the functions of T cells, B cells and NK cells. Moreover, Kyn and its’ downstream metabolites can activate aryl hydrocarbon receptor (AHR), which will result in the creating the immunosuppressive microenvironment (Huang et al., 2022; Zhang et al., 2022). The second is the GCN2 pathway. General control nonderepressible 2 (GCN2) is a serine/threonine-protein kinase (Munn et al., 2005). There is a domain in GCN2 that binds to uncharged tRNA to sense amino acid deficiencies. When the level of Trp in cells is low, uncharged tRNAs accumulate in cells and activate GCN2. The activated GCN2 eventually reduces the proliferation of T cells and promotes the differentiation of Treg cells. The third is the mechanistic target of rapamycin complex 1 (mTORC1) pathway. mTORC1 regulates various cellular process (Metz et al., 2012). The level of amino acids strongly affects mTORC1 activity. When the Trp level is decreased, mTORC1 is inhibited, which eventually induces T cell autophagy.

### 2.2 Inflammation

Inflammation is a normal response to injury or infection and is initiated by the innate immune system to clear away damaged cells (Medzhitov, 2008; Sorgdrager et al., 2019; Esmaeili and Hajavi, 2022). The hallmark of inflammation is the accumulation of various primary inflammatory cells in tissues, which overexpress various cytokines, growth factors, and enzymes. Thus, inflammation is crucial in maintaining health. However, if the tissue remains inflamed for a long time, inflammation can also damage healthy tissue and induce secondary repair, including fibrosis. Proinflammatory cytokines, such as interferon gamma, and other inflammatory signaling molecules, including the lipid mediator prostaglandin E2 and lipopolysaccharide pathogen particles, induce the overexpression of IDO1 (Baumgartner et al., 2019; Alves de Souza et al., 2022). The activation of IDO1 in response to these inflammatory factors induces immune tolerance and eventually controls hyperinflammation (Zhai et al., 2020). The mechanism of IDO1 involvement in inflammation regulation occurs *via* two main pathways (Heidari et al., 2020; Ogbechi et al., 2020; Gargaro et al., 2022). In the first, the overexpression of IDO1 consumes and depletes the intracellular Trp, thereby mediating immune tolerance. Several metabolites of IDO1 are known to toxic to immune cells and inhibit the regular functions of various immune cells (Huang et al., 2022; Zhang et al., 2022). In the second, the overexpression of IDO1 causes the accumulation of Kyn, which activates the aryl hydrocarbon receptor. This IDO1/Kyn/aryl hydrocarbon receptor signaling pathway regulates T cell activation, induces the differentiation of Treg cells, and changes the immunogenicity of antigen-presenting cells, which eventually has an anti-inflammatory effect (Romani et al., 2008; Sorgdrager et al., 2019). Interestingly, an imbalance in the Kyn/Trp ratio is often observed in inflammation-related disease, including infections and autoimmune disorders (Schröcksnadel et al., 2006; Huang et al., 2020). In summary, IDO1 is overexpressed in response to inflammation and suppresses the immune system to control inflammation.

### 2.3 Depression

Depression is a mental disorder that has a complicated mechanism (Barreto et al., 2018). Although different hypotheses have been proposed to explain the pathophysiology of depression, the monoaminergic hypothesis, which proposes that depression stems from low levels of the monoamine serotonin (5-hydroxytryptamine) in the brain, has become the basis for developing antidepressant drugs (Healy and Leonard, 1987; Badawy and Morgan, 1991). Serotonin is produced from the metabolism of *L*-Trp. Less than 5% of *L*-Trp is processed to synthesize serotonin, and the other 95% of *L*-Trp in the plasma is consumed by IDO1 to produce Kyn. Trp levels are much lower in the brains of depressed patients than in non-depressed people, and the levels of Trp are clearly associated with the symptoms of depression (Badawy and Morgan, 1991; Platten et al., 2021). Furthermore, depressed patients have high plasma levels of pro-inflammatory cytokines, such as interferon gamma. In an animal model of depression, the activation of IDO1 and increased levels of Kyn have also been measured. Because IDO1 activity is closely related to Trp levels, the activation of IDO1 and the involvement of the immune system and inflammation in depression suggest that the inhibition of IDO1 could be a target for discovering antidepressant drugs (Romani et al., 2008; Huang et al., 2020).

## 3 Natural indoleamine 2, 3-dioxygenase 1 inhibitors

Because IDO1 is an important immune checkpoint modulator, it is important in tumor immune escape, and thus is an important therapeutic target in cancer therapy. Over the past decade, there has been intense interest in developing IDO1 inhibitors in both academic institutes and the pharmaceutical industry. A variety of IDO1 inhibitors have been found *via* methods including high-throughput screening, rational design, and natural compound screening (John et al., 2010). Natural compounds are an important source of pharmacological agents. In the early stages of IDO1 inhibitor discovery before 2010, natural compounds contributed much important structural information for the rational design of IDO1 inhibitors (Delfourne, 2012). Next, we summarize and analyze natural compounds derived IDO1 inhibitors.

### 3.1 Quinones

**FIGURE 4:**
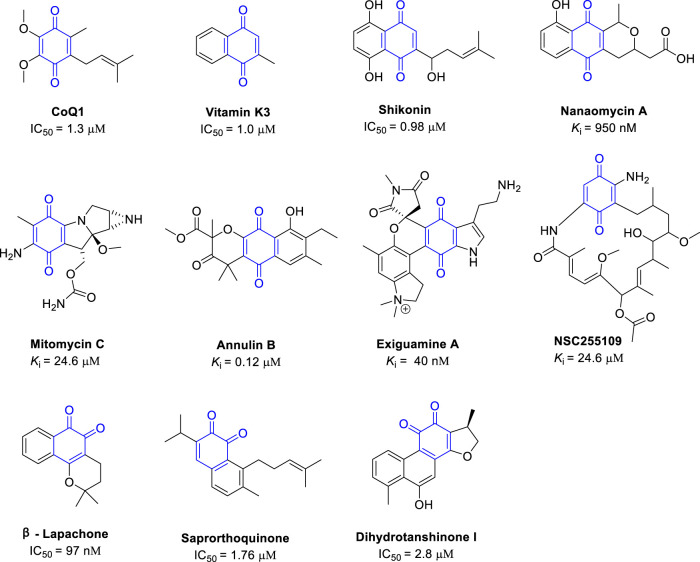
Structures of natural 1, 2- and 1, 4-quinones with their IDO1 inhibitory activity.

Natural quinones are classified by structure as benzoquinones, naphthoquinones, anthraquinones, and phenanthraquinones. According to the position of the carbonyl group, quinones can also be divided into 1, 2-quinones and 1,4-quinones (Zhang et al., 2021). Natural quinones were one of the first types of IDO inhibitors discovered (Figure 4) (Pereira et al., 2006), and most natural quinones have some IDO1 inhibitory activity. The quinone moiety usually occupies binding pocket A of the IDO1 active center, and can bind to the heme iron at the bottom of the pocket. In this part, we summarize the reported natural quinones with substantial IDO1 inhibitory activity, the structure of these quinones were shown in Figure 4.

Coenzyme Q (CoQ), also called ubiquinone, is a coenzyme family, that is, ubiquitous in cells and membranes in animals and bacteria and has important functions in cell metabolism, including in the mitochondrial respiratory chain (Turunen et al., 2004). CoQ1 is a member of the CoQ family and contains the core 1, 4-benzoquinone scaffold. CoQ1 is a moderate IDO1 inhibitor with an IC_50_ of 1.3 μM. Because CoQ1 is a simple quinone, it has been modified to improve the IDO1 inhibition activity (Ding et al., 2019). Another important natural quinone, vitamin K3, also called menadione, contains the naphthoquinone scaffold. Menadione has some important pharmacological functions, including the regulation of cell proliferation and cell growth, and has moderate IDO1 inhibitory activity with an IC_50_ of 1.0 μM (Kumar et al., 2008). However, substituting the methyl side chain in menadione with a long lipid chain to form vitamin K1 abrogates the IDO1 inhibitory activity. Due to their structure simplicity and activity, CoQ1 and menadione are suitable as lead compounds for further structural optimization to improve the IDO1 inhibitory activity.

Screening of natural compound libraries has revealed several natural compounds with interesting IDO1 inhibitory activity. Shikonin A, which is usually isolated from *Radix Arnebiae* and has anti-inflammatory activity attributed to the inhibition of caspase 1. Shikonin A inhibits IDO1 activity in a dose-dependent manner with an IC_50_ of 0.98 μM. Given the potency of IDO1 inhibition, it is likely that the anti-inflammatory activity of shikonin A also partially arises from IDO1 inhibition (Guo et al., 2020). The 1, 4-quinone antibiotic nanaomycin has been report to have the activity to inhibit DNA methyltransferase 3B (IC_50_ = 500 nM). Nanaomycin also found with the IDO1 inhibitory activity (*K*_i_ ∼ 950 nM) (Pantouris and Mowat, 2014). Lastly, the 1, 4-quinone mitomycin C also has moderate IDO1 inhibitory activity (*K*_i_ = 24.2 μM).

In 2006, the Andersen group found that the MeOH extract of the northeastern Pacific marine hydroid, *Garveia annulata* had IDO1 inhibitory activity. Further separation of the crude yielded a series of quinones with potent IDO1 inhibitory activity, of which annulin B was the most potent (*K*_i_ = 0.12 μM) (Pereira et al., 2006). This was the first reported natural IDO1 inhibitor. The Andersen group also collected the marine sponge *Neopetrosia exigua* in Papua New Guinea and found that the methanol extract had IDO1 inhibitory activity. The alkaloid exiguanine A isolated from the MeOH extract is one of the most potent natural IDO inhibitors (*K*_i_ = 41 nM) (Carr et al., 2008), and thus was selected to be optimized further (Dong et al., 2021). The Mowat group screened about 2800 natural compounds from the National Cancer Institute for IDO and TDO inhibitors and found several other natural compounds with potent IDO1 inhibitory activity. NSC255109 (17-aminodemethoxygeldanamycin) inhibits IDO1 with *K*_i_ of around 1.4 μM (Pantouris and Mowat, 2014), and this compound contains the 1, 4-benzoquinone scaffold as part of a cyclized structure.

Next, we describe IDO1 inhibitors with a 1, 2-quinone scaffold. *β*-Lapachone, which was first extracted from the lapacho tree (*Tabebuia avellanedae*), has anticancer activity, and the proposed mechanism is the activation of a non-caspase proteolytic pathway. However, *β*-lapachone is also a potent IDO1 inhibitor with an IC_50_ of around 97 nM (Medzhitov, 2008), and thus *β*-lapachone is also likely to alter the tumor immune environment, contributing to the clearance of tumor cells. Saprorthoquinone, which was isolated from the traditional Chinese medicine, Salvia prionitis Hance, is cytotoxic and inhibits IDO1 with an IC_50_ of 1.76 μM (Lin et al., 2020). Similarly, dihydrotanshinone I, which was isolated from the traditional Chinese medicine Radix Salviae Miltiorrhizae, is cytotoxic against many types of cancer cells and inhibits IDO1 with an IC_50_ of 2.8 µM (Guo et al., 2020).

In summary, from the structure-activity relationship of the quinones and IDO1, we found that 1, 2- and 1, 4-quinone core moieties bind well to the heme in the active center of IDO1. Due to the structural priority and potent IDO1 inhibitory activity, many efforts have been dedicated to optimize quinones derived inhibitors, and any further substitutions to the quinone moiety need to be careful evaluated so that they improve rather than disrupt the binding activity (Austin et al., 2014; Carvalho et al., 2014; Centko et al., 2014; Blunt et al., 2015; Shiokawa et al., 2016; Feng et al., 2018; Pan et al., 2018; Yang et al., 2018; Zhang et al., 2018; Zhao et al., 2019; Kong et al., 2020). Considering the IDO1 inhibitory potency of these quinones, their pharmacological benefits, such as anti-inflammatory and anticancer activities, may arise partially from their interaction with IDO1.

### 3.2 Polyphenols

**FIGURE 5:**
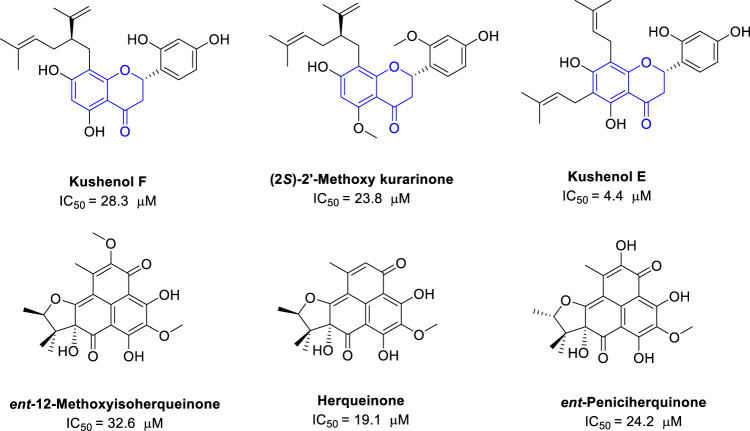
Structures of natural polyphenols with their IDO1 inhibitory activity.

Polyphenols are a large family of natural compounds with various interesting biological activities. Some polyphenols were also found with potential anti-IDO1 activities. The plant *Sophora flavescens*, which contains many flavonoids, is used in traditional Chinese medicine to treat cancers. Screening for IDO1 inhibitors identified three flavonoids isolated from S. *flavescens* with moderate inhibition activity. Kushenol E has the most potent IDO1 inhibitory activity with an IC_50_ of 4.4 μM, followed by (2*S*)-2′-methoxy kurarinone (IC_50_ = 23.8 μM) and kushnol F (IC_50_ = 28.3 μM) (Kwon et al., 2019) (Figure 5). In addition, a Korean group also reported the polyphenols from the Hawaiian volcanic associated fungus *Penicillium herquei* FT729. Among them, herqueinone has an IC_50_ of 19.1 μM against IDO1, peniciherquinone inhibits IDO1 with IC_50_ of 24.2 μM, and *ent*-12-methoxyisoherqueinone has an IC_50_ of 32.6 μM against IDO1 (Yu et al., 2022). So far, the polyphenols only reported with moderate IDO1 inhibitory activities.

### 3.3 Alkaloids

**FIGURE 6:**
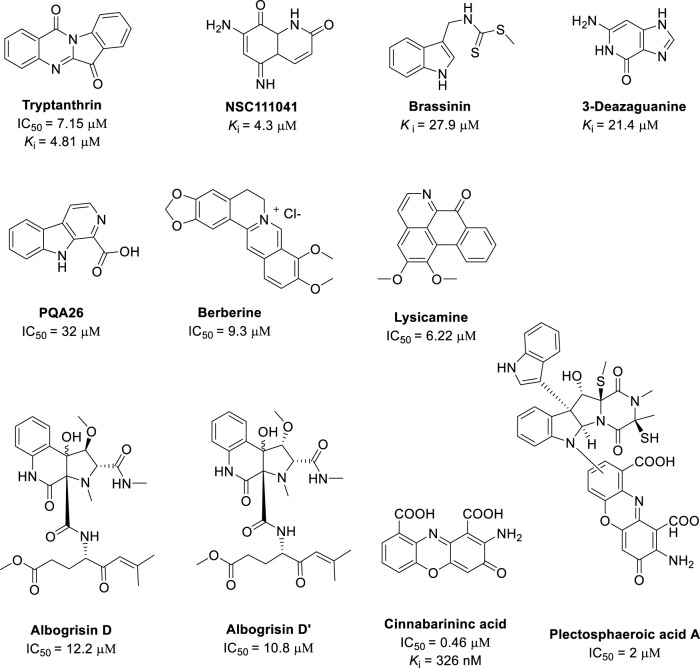
Structures of natural alkaloids with their IDO1 inhibitory activity.

Alkaloids are a structurally diverse class of naturally occurring bases containing at least one nitrogen atom. In this section, we list the alkaloids in the order of their structural simplicity (Figure 6). First, we describe two alkaloids that have some structural similarity to 1, 4-quinones. Tryptanthrin (indolo [2,1-b]quinazolin-6,12-dione) has been extracted from the Chinese medicinal plants *Polygonum tinctorium* and Isatis tinctoria, and it has various pharmacological activities, including cytotoxicity against several parasites and microorganisms, and it inhibits IDO1 with an IC_50_ of 7.15 μM (Yang et al., 2013). Tryptanthrin also has two carbonyl groups that point in opposite directions, which is structurally similar to 1, 4-quinones. In addition, NSC111041 was one of several natural compounds with interesting IDO1 inhibitory activity identified by the Mowat group among about 2800 compounds from the National Cancer Institute during a screening campaign for IDO and TDO inhibitors. NSC111041 inhibits IDO1 with *K*_i_ of 4.3 μM (Delfourne, 2012; Pantouris et al., 2014). In NSC111041, one of the quinone carbonyl groups is replaced with an imino group (Dolušić et al., 2013), and thus NSC111041 is structurally similar to 1, 4-quinones.

A library of indole analogues were screened for IDO1 inhibitors. Brassinin is an indole-based natural product with reported antifungal and anticancer activity, Brassinin has moderate to low IDO1 inhibitory activity (*K*_i_ = 97.7 µM) (Gaspari et al., 2006). Another indole-derived alkaloid is PQA26, which was isolated from the medicinal deciduous tree, *Picrasma quassioides* (D. Don) Benn, that is, widely grown in south China. The dry branches are used in traditional Chinese medicine for heat clearing, detoxification, and eliminating dampness. A virtual screening method suggested that PQA26 had IDO1 inhibition activity, and moderate IDO1 inhibitory activity (IC_50_ = 32 μM) was confirmed experimentally (Wang et al., 2019). 3-Deazaguanine was also identified as having IDO1 inhibitory activity in the micromolar range (*K*_i_ = 21.4 μM) (Pantouris and Mowat, 2014).

Berberine is a bitter, yellow natural compound, which is isolated from plants in the berberis genus and is an important ingredient in the traditional Chinese medicine, Oren-gedoku-to. Berberine can help to maintain a normal body weight and normal blood sugar levels. Furthermore, berberine has IDO1 inhibitory activity with an IC_50_ of 9.3 µM. Medicinal chemists have optimized the IDO1 inhibition activity of berberine further (Yu et al., 2010; Wang et al., 2018). Lately, a research group from China reported their isolation of fourteen novel and known alkaloids from the rhizomes of Sinomenium acutum. Among these alkaloids, lysicamine show an IDO1 inhibitory activity with IC_50_ values of 6.22 ± 0.26 μM (Bi et al., 2022).

Aminophenoxazinones are a group of natural dyes that includes actinomycines, which have antibiotic activity. Some aminophenoxazinones have IDO1 inhibitory activity. For example, cinnabarinic acid is a potent IDO1 inhibitor with an IC_50_ of 0.46 μM (Pasceri et al., 2013). Several alkaloids contain the cinnabarinic acid moiety. For example, plectosphaeroic acids A–C were isolated from the fungus *Plectosphaerella cucumerina*, which was cultured from marine sediment from Barkley Sound, British Columbia. These three alkaloids also contained the cinnabarinic acid moiety in their structures, and they are IDO1 inhibitors with an IC_50_ of 2 μM (Carr et al., 2009). The alkaloid stereoisomers albogrisin D and albogrisin D′ were isolated from *Streptomyces albogriseolus* MGR072 collected from a mangrove reserve in Fujian Province, China. These compounds have similar IDO1 inhibitory activities with IC_50_ values of around 10 μM (Gao et al., 2019).

### 3.4 Others

**FIGURE 7:**
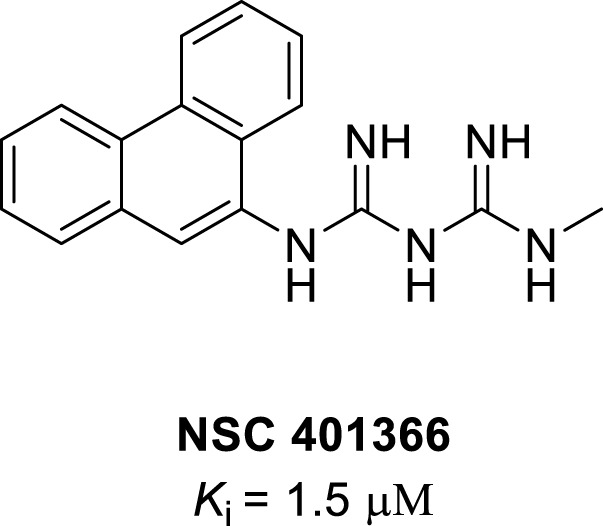
Structure of NSC401366.

NSC401366 (*N*-methyl-*N*″-9-phenanthrylimidodicarbonimidic diamide) is a natural anthracene compound with potent IDO1 inhibition activity, which has a different structure from other IDO1 inhibitors (Figure 7). NSC401366 was discovered by screening the methanol extracts of marine organisms and has potent IDO1 inhibitory activity with a *K*_i_ of 1.5 μM (Vottero et al., 2006).

## 4 Conclusion

The balance in the levels of Trp and Kyn in plasma regulates several important physiological process, such as immune activation and immune tolerance. IDO1 is a key metabolic enzyme responsible for the metabolism of Trp to Kyn. The activation of IDO1 changes the concentration ratio between Trp and Kyn, and thus IDO1 is closely related to several important physiological disorders, including cancer, inflammation, and depression. Due to the importance of IDO1, IDO1 inhibitors have been the focus of intense interest in the pharmaceutical industry (Chen et al., 2021). Several candidate IDO1 inhibitors (Platten et al., 2019; Chen et al., 2021), including indoximod, navoximod, epacadostat, and linrodostat, have entered the clinical research stage for cancer immunotherapy.

Nature is an important source from which many therapeutic agents are obtained. Traditional medicine exploits the unique mode of actions of natural compounds to mitigate functional disorders. Modern medicine is exploring the mechanisms of many traditional medicines to optimize the pharmacological activity of these traditional medicines and mitigate the side effects. This kind of research is difficult because of the complexity of the mechanisms and unknown combined effects. However, it is helpful to begin by demonstrating the modes of action of individual natural compounds. Based on the structure-activity information, scientists can understand and analyze the pharmacological benefits of traditional medicine.

Thus, in this review we have summarized the interactions between IDO1, an important metabolic enzyme, and a group of natural compounds that have IDO1 inhibition activity. We have listed all the natural compounds reported so far to have moderate to strong IDO1 inhibitory activity, and the quinones are the most promising of these compounds. This finding provides structure-activity information that will help medicinal chemists to understand the pharmaceutical benefits of natural compounds (Ianni et al., 2022) and to design potent IDO1 inhibitors.
